# Plasma concentration of voriconazole under concomitant use of nirmatrelvir/ritonavir in patients with COVID-19: a multicenter retrospective study

**DOI:** 10.3389/fphar.2025.1654671

**Published:** 2025-08-15

**Authors:** Hao Shi, Xuan Zhang, Qihui Kong, Lingyan Yu, Zhenwei Yu

**Affiliations:** ^1^ Department of Pharmacy, Shaoxing Campus, Sir Run Run Shaw Hospital, School of Medicine, Zhejiang University, Shaoxing, China; ^2^ Department of Pharmacy, Northern Jiangsu People’s Hospital, Yangzhou, China; ^3^ Department of Pharmacy, Qingchun Campus, Sir Run Run Shaw Hospital, School of Medicine, Zhejiang University, Hangzhou, China; ^4^ Department of Pharmacy, Second Affiliated Hospital, School of Medicine, Zhejiang University, Hangzhou, China; ^5^ Research Center for Clinical Pharmacy, Zhejiang University, Hangzhou, China

**Keywords:** nirmatrelvir/ritonavir, voriconazole, drug interaction, therapeutic drug monitoring, CYP = cytochrome P450

## Abstract

**Background:**

The concomitant use of nirmatrelvir/ritonavir and voriconazole is generally contraindicated in clinical practice because of drug‒drug interactions (DDIs). However, emerging clinical data suggest that this DDI is complicated and that the concomitant use of these two drugs may be feasible.

**Methods:**

This was a multicenter retrospective study. Hospitalized patients who were diagnosed with COVID-19 in 2023 and who received concomitant nirmatrelvir-ritonavir and voriconazole were retrospectively included according to preset criteria. Personal information, medication records and voriconazole plasma levels were obtained from the hospital information system. The voriconazole concentrations were analyzed.

**Results:**

A total of 13 hospitalized patients with COVID-19 and 16 voriconazole trough concentrations were included from 4 centers. Half of the patients (8 patients, 50.0%) had voriconazole plasma concentrations within the therapeutic range. The remaining 8 cases (50.0%) fell outside the therapeutic range, including 1 case (12.5%) with subtherapeutic levels and 7 cases (87.5%) with supratherapeutic concentrations.

**Conclusion:**

The concomitant use of nirmatrelvir/ritonavir and voriconazole might be feasible, but the dosing of voriconazole needs further study.

## 1 Introduction

Nirmatrelvir/ritonavir is an oral antiviral formulation for COVID-19 treatment that has been designated by the World Health Organization as the prefer therapeutic option for high-risk populations susceptible to severe disease progression (Agarwal et al., 2020). Ritonavir is a strong CYP3A4 inhibitor that can also induce CYP2C19; therefore, concomitant use of nirmatrelvir/ritonavir may lead to clinically significant drug‒drug interactions (DDIs). Fungal infection, such as invasive aspergillosis, is common in patients with severe COVID-19 (White et al., 2020). Voriconazole is one of the first-line options for severe fungal infections (Douglas et al., 2021). However, coadministration of nirmatrelvir/ritonavir with voriconazole is contraindicated in product labeling and widely discouraged in therapeutic guidelines (Merck, 2022). The pharmacokinetic interaction between nirmatrelvir/ritonavir and voriconazole exhibits significant mechanistic complexity. Expected interactions stem from ritonavir’s potent inhibition of CYP3A4, which may theoretically induce significant increases in systemic exposure. Unexpected interactions arise from ritonavir-induced CYP2C19 enzyme induction during prolonged use (>7 days), leading to unanticipated late-phase concentration reductions, combined with nirmatrelvir’s competitive inhibition that exacerbates nonlinear pharmacokinetics (Wang et al., 2024). Although the interaction between nirmatrelvir/ritonavir and voriconazole is rather complex, recent evidence suggests that their combination is feasible (Wang et al., 2023). Thus, we performed this multicenter retrospective study to assess the plasma level of voriconazole under the concomitant use of nirmatrelvir/ritonavir in patients with COVID-19.

## 2 Methods

### 2.1 Patient inclusion

This study was a multicenter retrospective study. Inpatients meeting the following criteria were included in the study: (1) hospitalized patients due to COVID-19 infection in 2023; (2) patients aged over 18 years; (3) patients receiving concomitant nirmatrelvir-ritonavir and voriconazole; and (4) patients with available voriconazole plasma levels during concomitant use. Owing to the irreversible inhibitory effect of ritonavir on CYP3A4, patients receiving voriconazole treatment within 5 days of nirmatrelvir-ritonavir withdrawal were also considered to have potential DDI risk and were included.

### 2.2 Data collection and analysis

The following personnel information and drug administration information were extracted from the hospital information system: patient age, sex, body weight and height; admission to the ICU or receiving organ support, such as CRRT and ECMO; laboratory test results indicating renal and hepatic function; formulation, dose, dosing frequency and treatment length of both drugs; and therapeutic drug monitoring (TDM) of voriconazole.

A voriconazole concentration of 1–5.5 mg/g was considered the therapeutic range (Ashbee et al., 2014). The data are descriptively presented. Continuous data are shown as medians and IQRs, and categorical data are shown as numbers and percentages. The voriconazole concentrations were stratified by dose, formulation, and other meaningful variables.

## 3 Results

A total of 13 hospitalized patients were included from four centers. The demographic characteristics are shown in Table 1. Most patients were old, and four were admitted to the ICU. All the patients received voriconazole at 12-h intervals, but only four patients received a reduced maintenance dose. Among these patients, 5 (38.4%) received oral voriconazole, 4 (30.8%) received intravenous voriconazole, and 4 (30.8%) underwent sequential therapy during nirmatrelvir/ritonavir treatment. Nine patients (69.2%) received a maintenance voriconazole dose of 200 mg every 12 h, whereas 4 (30.8%) received a reduced dose of 100 mg every 12 h.

**TABLE 1:** Characteristics of included patients.

Variable	Overall (n = 13)
Age, y, median (IQR)	68.0 (58.0, 72.0)
Male, n (%)	10 (76.9)
Weight, kg, median (IQR)	67.0 (50.5, 74.5)
Height, cm, median (IQR)	170.0 (162.0, 173.0)
Body surface area, m^2^, median (IQR)	1.76 (1.47, 1.84)
BMI, median (IQR)	22.9 (18.6, 25.6)
Laboratory data, median (IQR)	
ALT, U/L	35.0 (28.0, 111.8)
AST, U/L	37.0 (23.8, 137.3)
ALP, U/L	87.5 (71.8, 160.0)
TBiL, U/L	13.4 (8.6, 19.7)
Serum creatinine, µmol/L	80.5 (50.5, 101.5)
Organ support treatment, n (%)	
Continuous Renal Replacement Therapy	3 (23.1)
Extracorporeal Membrane Oxygenation	1 (7.7)
Admitted to the ICU	4 (30.8)
Administration route Voriconazole treatment, n (%)	
Oral	5 (38.4)
Iv	4 (30.8)
Sequential Therapy	4.0 (30.8)
Voriconazole maintenance dose, n (%)	
100 mg	4 (30.8)
200 mg	9 (69.2)

ALT, alanine transaminase; AST, aspartate aminotransferase; ALP, alkaline phosphatase; TBiL, total bilirubin; Oxygenation; Oral, Oral Administration; Iv, Intravenous Injection; IQR, interquartile range; BMI, body mass index; ICU, intensive care unit.

A total of 16 voriconazole plasma concentration values were obtained. Six of these concentrations were used concomitantly, and 10 were used after nirmatrelvir/ritonavir withdrawal. All post-use plasma concentrations were measured within 3 days. Half of the voriconazole concentrations were within the reference range. One patient even had a low voriconazole concentration during concomitant use. The detailed voriconazole concentrations are shown in Table 2. The rate of achieving the therapeutic window for intravenous voriconazole was 60% (3/5), and for oral administration, it was 40% (2/5); for the 100 mg dose of voriconazole, the rate within the therapeutic window was 50% (2/4), and for the 200 mg dose, it was 50% (6/12); the rate within the therapeutic window during nirmatrelvir/ritonavir treatment was 66.7% (4/6); and after nirmatrelvir/ritonavir treatment, it was 40% (4/10).

**TABLE 2:** Concentration distribution of voriconazole under the concomitant use of nirmatrelvir/ritonavir.

Voriconazole concentration	Result
Overall, median (IQR) μg/mL	4.8 (3.5, 7.6)
within the therapeutic range, n (%)	8 (50.0)
above the therapeutic range, n (%)	7 (87.5)
below the therapeutic range, n (%)	1 (12.5)
Subgroups^a^	
Iv	60.0 (3/5), 4.0 (3.6, 9.3)
Oral	40.0 (2/5), 6.3 (3.6, 7.5)
100 mg maintenance dose	50.0 (2/4), 5.0 (3.6, 7.3)
200 mg maintenance dose	50.0 (6/12), 4.8 (3.4, 7.6)
During nirmatrelvir/ritonavir use	66.7 (4/6), 3.4 (2.7, 3.5)
After nirmatrelvir/ritonavir withdraw	40.0 (4/10), 6.9 (4.4, 7.7)

The therapeutic range of voriconazole is set as 1–5.5 μg/mL. IQR, interquartile range.

^a^ Date were presented as number within the therapeutic range (%), and median concentration (IQR) in μg/mL.

## 4 Discussion

This study provides multicenter clinical evidence of voriconazole concentration distributions under the concomitant use of nirmatrelvir/ritonavir. Half of the concentrations were within the therapeutic range. These findings may support the feasibility of nirmatrelvir/ritonavir and voriconazole coadministration, which may be important during epidemics and for source-limiting settings. Moreover, ritonavir is a common CYP booster in many combinations, and the results of this study would also be useful for handling DDI between voriconazole and other ritonavir containing formulations.

Although data from 4 centers were retrospectively analyzed, only 13 patients were included. These findings indicate that the DDI between nirmatrelvir/ritonavir and voriconazole were well known and avoided in clinical practice (Marzolini et al., 2022). However, ritonavir has irreversible inhibitory effects on CYP3A4, and the DDI risk can last several days after nirmatrelvir/ritonavir withdrawal. In this study, 10 of the 16 plasma concentrations were obtained after the withdrawal of nirmatrelvir/ritonavir, which indicated that this post-use DDI risk was neglected in clinical practice (Han et al., 2025). This study revealed that among 13 COVID-19 patients coadministered nirmatrelvir/ritonavir and voriconazole, 50% (8/16) of the monitored voriconazole plasma concentrations remained within the therapeutic range (1–5.5 μg/mL). A large cohort including 597 trough concentrations in Chinese patients found that 65.0% of the concentrations are within the target therapeutic range (1–5 μg/mL), while the proportions <1 mg/L and >5 mg/L were 11.7% and 23.3%, respectively (Wang et al., 2022). Another retrospective study in US analyzing the target achievement of voriconazole had included 250 concentrations, and found that 54% of concentration are therapeutic (reference range 1–5 μg/mL), 26% were subtherapeutic and 20% were super therapeutic (Yi et al., 2017). Although it is difficult to make a statistical comparison, we can find that patients with concomitant nirmatrelvir/ritonavir had similar percentages in therapeutic range achievement, but higher in percentages in super therapeutic concentrations. And this needs further validation. These findings indicate that the concomitant use of nirmatrelvir/ritonavir and voriconazole may be feasible, which is different from current label information and clinical guidelines. Although numerous studies have reported adverse outcomes when nirmatrelvir/ritonavir is combined with drugs metabolized by CYP3A4, this phenomenon is a reasonable explanation (Haque et al., 2023; Qin et al., 2023). As a pharmacokinetic booster, ritonavir inhibits CYP3A4 to slow the metabolism of nirmatrelvir, as do other drugs metabolized by CYP3A4. This effect is always overlooked in clinical practice. Notably, concomitant use of voriconazole should be avoided on its label because of the concern of a low concentration of voriconazole. A previous study focusing on the influence of a single dose of ritonavir also revealed that the concentrations of voriconazole were elevated (Mikus et al., 2006). Wang et al. reported that the voriconazole concentration may be greater or lower in patients with COVID-19 when coadministered with nirmatrelvir/ritonavir, and the difference may be attributed to polymorphisms of the CYP2C19 genotype (Wang et al., 2023). Although the evidence of DDI between voriconazole and nirmatrelvir/ritonavir in COVID-19 patients is limited, the DDI between voriconazole and ritonavir (or its combination with other antiviral drugs) has been reported. Zhu et al. reported that when coadministered with atazanavir-ritonavir in healthy subjects, voriconazole exposure decreased in CYP2C19 extensive metabolizers but increased in CYP2C19 poor metabolizers (Zhu et al., 2017). Liu et al. reported that after a 10-day duration of ritonavir treatment, voriconazole exposure decreased to different extents, depending on the ritonavir dose (López-Hernández et al., 2025).

Additionally, we investigated voriconazole plasma concentrations among different subgroups, which were stratified on the basis of formulation, maintenance dose and concomitant course. Intravenous voriconazole demonstrated a 60% probability (3/5) of concentrations within the therapeutic range versus 40% (2/5) for oral administration. This is very reasonable, as oral voriconazole has high bioavailability (Harada et al., 2021). The observed reduction in therapeutic attainment with the oral formulation (40% vs 60% IV) aligns with established pharmacokinetic properties of voriconazole: Significantly reduced oral bioavailability attributable to CYP450-mediated metabolism in the gut/liver. The dose significantly influences voriconazole exposure; however, both the 100 mg and 200 mg maintenance regimens yielded 50% therapeutic target attainment (2/4 and 6/12, respectively) and similar concentrations. This similar therapeutic target attainments between different dosing may be attributed to high inter-individual viability of voriconazole pharmacokinetic in patients and the limited sample size. A recent physiologically based pharmacokinetic modeling study suggested that ritonavir could increase voriconazole exposure to CYP2C19 intermediate and poor metabolizers rather than decrease it (Wang et al., 2024). Our results were in accordance with these studies. When we focused on the voriconazole plasma concentrations after discontinuation of nirmatrelvir/ritonavir, the concentrations were still high. CYP3A4 activity may need approximately 4 days to be restored, according to recent reports (Wang and Chan, 2022). Thus, the post-use DDI risk should be considered with caution.

The main limitation of this study is the small sample size. The genotypes of CYP3A4 and 2C19 in the included patients were unknown. Voriconazole concentrations when it is used alone were not collected and it is unable to make a direct comparison. The DDI was not quantified by advanced models, such as physiological-based pharmacokinetic models, which should be evaluated in the future.

In conclusion, the concomitant use of voriconazole and nirmatrelvir/ritonavir may be feasible, but the dosing of voriconazole under concomitant use needs further study.

## Data Availability

The original contributions presented in the study are included in the article/supplementary material, further inquiries can be directed to the corresponding authors.
